# Design of a fluorescent and clickable Ag_38_(SRN_3_)_24_ nanocluster platform: synthesis, modeling and self-assembling †

**DOI:** 10.1039/d1na00090j

**Published:** 2021-04-07

**Authors:** Gaetano Campi, Lorenza Suber, Giuliana Righi, Ludovica Primitivo, Martina De Angelis, Daniela Caschera, Luciano Pilloni, Alessandra Del Giudice, Amedeo Palma, Mauro Satta, Alessandro Fortunelli, Luca Sementa

**Affiliations:** CNR-Istituto di Cristallografia Via Salaria km 29,300-00015 Monterotondo Scalo Rome Italy gaetano.campi@ic.cnr.it; CNR-Istituto di Struttura della Materia Via Salaria km 29,300-00015 Monterotondo Scalo Rome Italy lorenza.suber@ism.cnr.it; CNR-IBPM-c/o Dip. Chimica, Sapienza Università di Roma p.le A. Moro 5 00185 Rome Italy; Dip. Chimica, Sapienza Università di Roma p.le A. Moro 5 00185 Rome Italy; CNR-Istituto per lo Studio dei Materiali Nanostrutturati Via Salaria km 29,300-00015 Monterotondo Scalo Rome Italy; ENEA SSPT-PROMAS-MATPRO, Materials Technology Division, Casaccia Research Centre 00123 Rome Italy; CNR-Istituto di Chimica dei Composti Organometallici Via G. Moruzzi 1 56127 Pisa Italy; CNR-Istituto per i Processi Chimico Fisici Via G. Moruzzi 1 56127 Pisa Italy luca.sementa@pi.ipcf.cnr.it

## Abstract

Fluorescent atomically precise Ag_38_(11-azido-2-ol-undecane-thiolate)_24_ nanoclusters are easily prepared using sodium ascorbate as a “green” reducer and are extensively characterized by way of elemental analyses, ATR-FTIR, XRD, SAXS, UV-vis, fluorescence spectroscopies, and theoretical modeling. The fluorescence and the atomically determined stoichiometry and structure, the facile and environmentally green synthesis, together with the novel presence of terminal azido groups in the ligands which opens the way to “click”-binding a wide set of molecular species, make Ag_38_(11-azido-2-ol-undecane-thiolate)_24_ nanoclusters uniquely appealing systems for biosensing, recognition and functionalization in biomedicine applications and in catalysis.

## Abstract 2

Fluorescent Ag_38_(SRN_3_)_24_ nanoclusters with the novel presence of terminal azido groups for easily “click”-binding a wide set of molecular species and their self-assembling in lamellar and hexagonal superstructures permeated by nanoregion defects.

## Graphical abstract

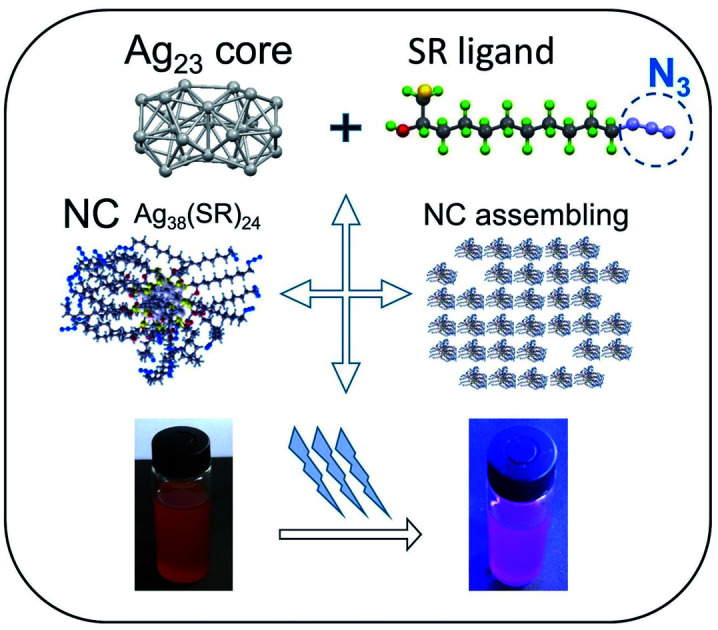

## 1. Introduction

Recently, thiolate-protected noble metal nanoclusters (noble metal = Au, Ag), also called “monolayer protected clusters” (MPC)^1,2^ or “atomically precise noble metal thiolate nanoparticles”^3,4^ because, differently from noble metal thiolate nanoparticles, they are composed of a precise number of noble metal atoms and thiolate molecules, have emerged as a new type of nanomaterials due to their distinctive physical and chemical properties. They can be denoted as M_*n*_(SR)_*m*_, where *n* and *m* are integers representing the number of noble metal atoms and ions (M^0^ + M^+^) and thiolate ligands (SR) respectively. Compared to noble metal thiolate nanoparticles, thiolate noble metal nanoclusters (NCs) have a hybrid nature, metallic and molecular,^5^ showing new properties suitable for applications in biomedicine,^6–9^ sensors and imaging,^10,11^ light energy conversion,^12,13^ catalysis^14–16^*etc.* In particular, in biomedicine, they could be used as biomolecule carriers, once the thiolate ligand is properly functionalized to bind biomolecules. To this aim, we have prepared the thiolate ligand with a terminal azido group able to bind molecules with carbon–carbon triple bond *via* “click” chemistry, a method today largely employed in chemistry mainly because it is reliable, specific and biocompatible.^17^

Due to the strong quantum confinement effects in the sub-2 nm size regime, M_*n*_(SR)_*m*_ have discrete electronic states and exhibit some unique molecule-like properties such as quantized charging,^18,19^ molecular chirality,^20,21^ and photoluminescence.^10,22–26^ The properties are highly dependent on the composition and structure of the M_*n*_(SR)_*m*_. For this reason, it is important to precisely control their composition at the atomic level. In the past few years, there have been many successful attempts in single-crystal X-ray structure determination of atomically precise Au_*n*_(SR)_*m*_,^27–31^ far less for Ag_*n*_(SR)_*m*_^23,32–36^ due to the higher reactivity of silver toward oxidation. The difficulty in the preparation mainly consists in setting up the conditions to form and stabilize the atomically precise M_*n*_(SR)_*m*_. As a matter of fact, for the preparation, a polymeric noble metal thiolate complex, (M^+^)_*x*_(SR)_*y*_, is used as the reagent and the reaction consists of a partial reduction of M^+^ ions by addition of an excess of a strong reducer, usually NaBH_4_.^5^ The reduction is immediate, as evidenced, for Ag_*n*_(SR)_*m*_, by a color change of the reaction dispersion from pale yellow to red-brown. Then usually an ageing (Ostwald ripening) and separation process are necessary to isolate the stable(s) Ag_*n*_(SR)_*m*_. In recent years, high-resolution separation techniques have helped to isolate atomically precise M_*n*_(SR)_*m*_.^37^

The main effort, however, is to find preparation methods able to control the reduction in order to avoid or reduce the time consuming separation processes and possible degradation of the products. Once prepared, the following steps would be: (a) crystallize the M_*n*_(SR)_*m*_ nanocluster and solve the single crystal X-ray structure, (b) correlate the NC structure to its chemical–physical properties using modeling and simulation techniques. In this way, the preparation of new M_*n*_(SR)_*m*_ would no longer depend only on a trial-and-error method, but on a precise design of the stable M_*n*_(SR)_*m*_ having the desired properties.

To address these goals, in the present work we have developed an original preparation protocol based on a milder reduction agent, sodium ascorbate, instead of sodium borohydride. The use of sodium ascorbate ensures a more selective but still efficient reduction process which, importantly, maintains the integrity of sensitive groups such as the azido ones, leading to a facile and massive production of a size-selected Ag_38_(11-azido-2-ol-undecane-thiolate)_24_ MPC that exhibits azido groups in terminal positions. We demonstrate that we have achieved the synthesis of the title compound *via* an extensive ATR-FTIR, XRD, SAXS, UV-vis, and fluorescence spectroscopic characterization, combined with theoretical simulation of the atomistic structure (*via* Global Optimization, GO, tools), optical response (*via* Time-Dependent Density-Functional Theory, TD-TDFT), and assembling (*via* Molecular Dynamics, MD). X-ray scattering measurements suggest the self-assembling of disordered crystalline domains in supramolecular lamellar and hexagonal phases permeated by nanoregion defects at nanoscale.

The greener and milder character of sodium ascorbate as a reducing agent represents an additional benefit of the approach here proposed. This achievement opens the way to the controlled synthesis of new nanoclusters to be applied as “molecule carriers” or “molecule supports” in many different fields varying from biomedicine to biosensing to catalysis.

## 2. Results and discussion

Usually noble metal thiolate nanoclusters are formed by reduction of the noble metal thiolate complex [(M^+^)_*x*_(SR)_*y*_] (M = noble metal, SR = thiolate), with the strong reducer NaBH_4_. In our case, by using NaBH_4_, also the azido group N_3_ of the SRN_3_ thiolate is reduced forming the amino group NH_2_. We have then employed a milder and “green” reducer such as sodium ascorbate. The reaction is not immediate as in the case of the reducer NaBH_4_ requiring longer reaction times and a higher temperature. Elemental C, H, N, S analyses are in agreement with the nanocluster formulation Ag_38_(SRN_3_)_24_. It is to note that the single crystal X-ray structure of Au_38_(SR)_24_ with R = C_2_H_4_(C_6_H_5_) has been solved^27^ and from literature it is also known the same structure where some Au atoms have been substituted with Ag atoms.^38^

**Fig. 1:**
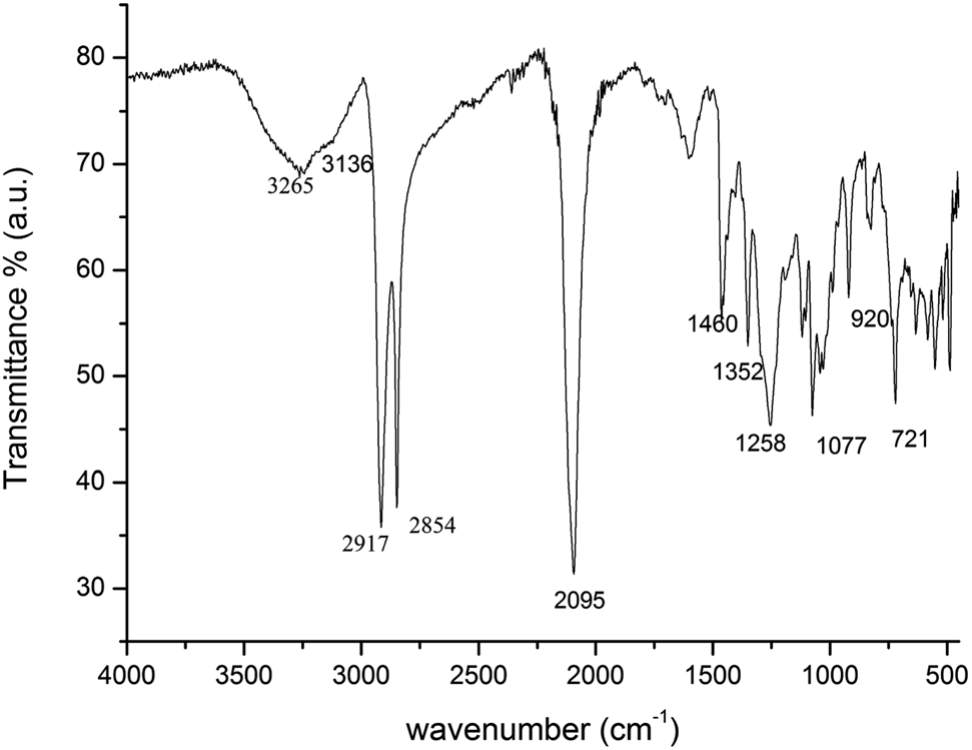
ATR-FTIR spectrum of Ag_38_(SRN_3_)_24_ NCs.

Fig. 1 is shown the ATR-FTIR spectrum of the Ag_38_(SRN_3_)_24_, presenting all the bands characteristic of the CH, OH and N_3_ groups of the 11-azido-2-ol-undecanethiolate; the broad band in the region 3300–3100 cm^−1^ is due to the O–H stretching vibration, the strong bands at 2917 and 2854 cm^−1^ to the C–H stretching vibrations whereas the strong and sharp band at 2095 cm^−1^ indicates the stretching vibration of the N=N=N azido group. The methylene/methyl band at 1460 cm^−1^ plus a sharp band at 721 cm^−1^ (methylene rocking vibration) is indicative of a long-chain linear aliphatic structure (note that the splitting observed for 1460 and 721 cm^−1^ bands is attributed to crystallinity and a high degree of regularity for the linear backbone structure).^39^ Moreover, the sharp band at 1352 cm^−1^ is attributable to the OH bending vibration, the band centered at 1258 cm^−1^ to the C–N stretching vibration and the sharp band at 1077 cm^−1^ to the C–O stretching vibration of the secondary alcohol.

**Fig. 2:**
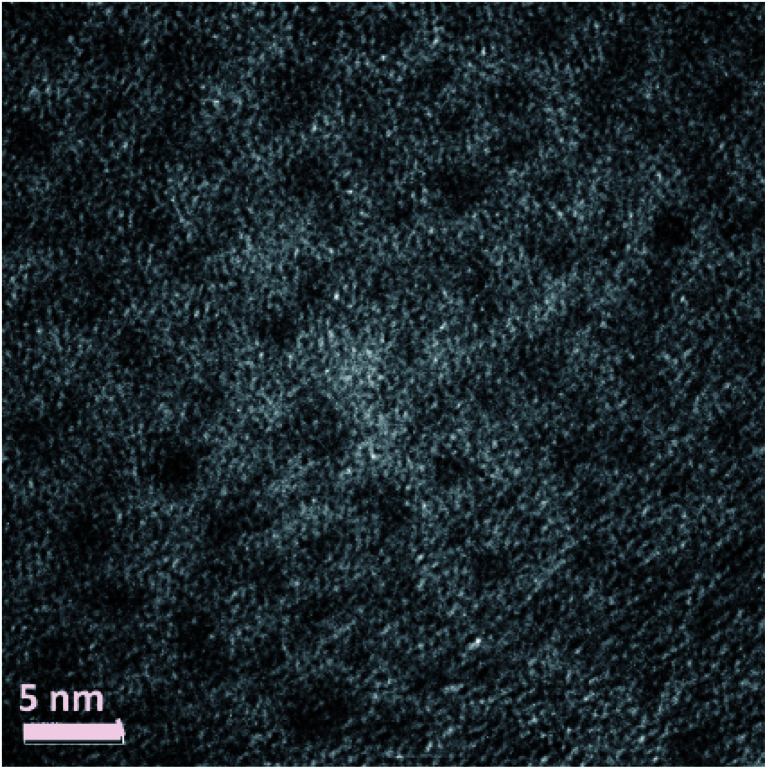
TEM image of Ag_38_ (SRN_3_)_24_ NCs.

In Fig. 2 is shown a TEM image of Ag_38_(SRN_3_)_24_ NCs displaying a diameter of about 3 nm.

### 2.1 Optical and spectroscopic properties (UV-vis and fluorescence)

**Fig. 3:**
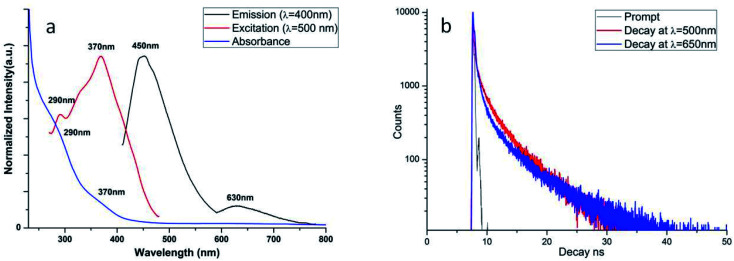
(a) UV-vis and fluorescence measurements (emission and excitation) for Ag_38_SR_24_; (b) time-resolved fluorescence measurements for Ag_38_(SRN_3_)_24_ at different wavelengths.

The optical properties of Ag_38_(SRN_3_)_24_ have been investigated and the results are shown in Fig. 3a.

The UV-vis spectrum (blue line) shows the presence of at least two different absorption signals, at 290 and 370 nm. These peaks can be attributed to the NC, as already observed in similar systems.^5^ No peaks, related to free silver nanoparticles, are visible in the range 420–460 nm. The excitation measurements are in good accordance with the absorption data. In particular, in Fig. 3a, the excitation spectrum (red line) of Ag_38_(SRN_3_)_24_ registered at 500 nm in the 270–480 nm range is reported as example. Two peaks at 290 nm and 370 nm are easily recognizable completely superimposable with those in the UV-vis spectrum.

The steady-state fluorescence (black line), measured exciting the solution at 400 nm, shows the existence of two different emission signals, one in the visible region of the spectrum at about 450 nm and the other in the IR region centered at 630 nm, lower in intensity with respect to the UV-vis one.

The same behavior has already been observed for similar silver thiolate NCs systems.^5^ Time-resolved fluorescence measurements (Fig. 3b) show that the fluorescence decays, collected at two different emission wavelengths, have a very similar profile. The deconvolution of the fluorescence decay profile collected at 650 nm has been resolved using three decay times (fit with *χ*^2^ = 1.6) with *τ*_1_ = 0.8 ns (relative population of *B*_1_ = 30%), *τ*_2_ = 5.1 ns (relative population of *B*_2_ = 32%) and *τ*_3_ = 0.01 ns (relative population of *B*_3_ = 38%). Very similar decay times have been calculated for the fluorescence decay at 500 nm, but the presence of the laser source at 405 nm, very close to the emission wavelength at which the decay has been measured, has the effect of amplifying the relative population for the shortest decay time (*B*_3_ up to 60% for *τ*_3,_ measuring at *λ* = 500 nm). Nevertheless, the spectroscopic measurements confirm that in the system there is only one emitting chemical species, with two different fluorescence emission signals, both of them related to Ag_38_(SRN_3_)_24_. These decay times are not surprising for these systems, already measured in silver nanoclusters with less than 25 silver atoms in the core.^40,41^ The fluorescent behavior of these nanoclusters is strongly influenced by many factors, such as the cluster size, the ligand nature and the steric effect of the shell on the metal core. Bigger Ag nanoclusters and/or the presence of a larger polydispersity can lead to a quenching effect in fluorescence, increasing decay times (in the order of ms).^42^ For other silver nanoclusters with characteristics similar to the Ag_38_(SRN_3_)_24_, the shorter lifetime decay has been attributed to the emission of a charge transfer state, while the longer one could correspond to the emission of the Ag core.^43,44^

The luminescence quantum yield measured for Ag_38_(SRN_3_)_24_ is 0.21, relative to Rhodamine 6G. This value is in good accordance with quantum yields of other silver nanoclusters systems^45,46^ and is quite a high value, making the emission readily observable under weak irradiation conditions, such as those available from laboratory UV lamps (see the observed emission presented in the Table of contents – graphical abstract, for example).

The fluorescent NCs with their terminal azido groups can, by way of “click” chemistry, using consolidated synthetic protocols, bind organic molecules,^47^ exploiting these systems in a multitude of applications, *e.g.*, as fluorescent carriers or markers in bio-sensing, recognition, in catalysis and biomedicine. In particular, experiments are underway to apply the NCs as catalyst-carriers in asymmetric catalysis. The NCs have been successfully functionalized by way of “click” chemistry with a chiral β-amino alcohol ligand, providing the corresponding nanostructured chiral catalyst; preliminary results employing the nanostructured catalyst in the Henry reaction are promising in terms of product yield and asymmetric induction, totally comparable with the homogeneous phase and, in addition, ease of catalyst recovery and recycling.^48^

### 2.2 X-ray scattering

**Fig. 4:**
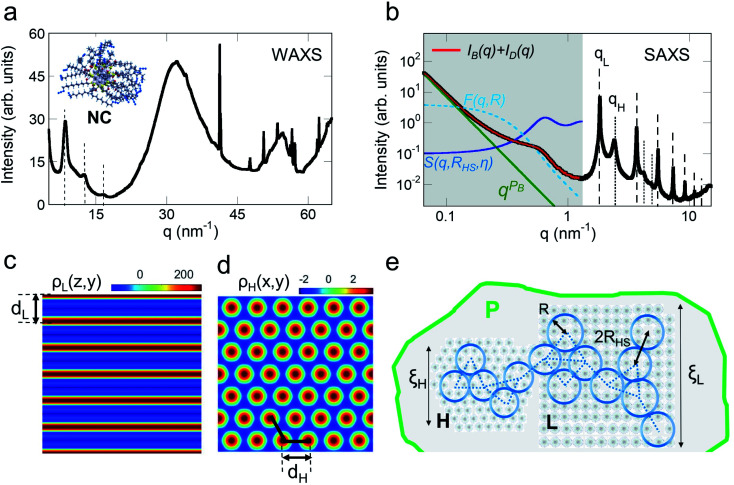
(a) Typical WAXS profile, *I*(*q*), of the sample measured on the XRD1 beamline at Elettra. We get evidence of sharp peaks due to diffraction spot reflections lying on concentric rings in the 2D frame. The peaks indicated by dashed lines of an ordered nanoscale phase are also shown. The inset represents the NC structure, calculated by DFT analysis, where the internal core and the external chains are visible. (b) SAXS profile, *I*(*q*), showing periodic peaks indicating (dashed lines) lamellar, L, and (dotted lines) hexagonal planar phases for *q* > 1.2 nm^−1^. At lower *q*-values, highlighted by the gray area, the (dashed light blue line) scattering amplitude *F*(*q*,*R*) and the (continuous blue line) structure factor *S*(*q*,*R*_HS_,*η*) for interacting hard spheres are reported, alongside the (green line) Porod law behavior due to larger particles P. The red line indicates the best-fitted curve obtained by the model described in ESI.† Electron density map of the (c) lamellar and (d) hexagonal phase. NCs assemble in the lamellar and hexagonal supramolecular structures with unit cell parameters *d*_L_ and *d*_H_, respectively. The red areas in the maps correspond to the NC cores with higher electron density. (e) Pictorial view of the NC assembly in the two phases H and L, aggregating to form large particles (P) permeated by the correlated defects network. A sphere with radius *R* represents each defect and the defects network is indicated by the dotted lines, where each dotted line is the defect correlation distance 2*R*_HS_. The size of the two phases is indicated by the coherent length *ξ*_H_ and *ξ*_L_.

Small fragments of our samples with sizes around 20 μm have been characterized by synchrotron X-ray diffraction on the XRD1 beamline at ELETTRA, Trieste.^49^ We obtained diffraction spots lying on weak concentric rings indicating that the sample is composed of an assembly of differently oriented crystals. A typical WAXS profile is shown in panel (a) of Fig. 4, where *q* is the momentum transfer *q* = 4π sin(*θ*)/*λ* with scattering angle 2*θ*, and X-ray energy wavelength *λ*. Indeed, we observe sharp peaks due to the sparse spots on rings alongside broad peaks ascribed to disorder generated by amorphous components or defects or overlapping of the diffraction peaks due to the anisotropic shape of the nanocrystals (*e.g.* long rod or extended platelets). The sharp peaks, due to NCs, composed of cores and polymeric chains (see the inset of panel (a)), do not correspond to any crystalline phase in the JCPDS PDF-2 database. Anyway, the WAXS patterns presented diffraction peaks towards lower values of *q* (dashed lines) as signatures of supramolecular order occurring at nanoscale.

Thus, to get further insight on the structure at nanoscale we performed Small Angle X-ray Scattering measurements. A typical SAXS *I*(*q*) profile is shown in panel (b) of Fig. 4. At lower *q*-values, *q* < 0.2, the SAXS profile, *I*(*q*), shows a Porod behavior ∼ *q*^−4^ indicating the formation of large particles (P). At higher *q*-values we can clearly observe quasi Bragg peaks at 
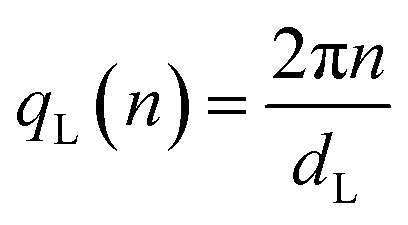
 up to order *n* = 7 (black dashed lines) due to a paracrystalline multilamellar phase, L, where *d*_L_ = 3.4 nm is the mean interlamellar separation.^50–53^ The average size of these lamellar phases can be determined from the Debye–Sherrer formula applied at the lamellar peaks and results to be around 130 nm. Beyond this main lamellar phase, we clearly observe three peaks corresponding to a planar hexagonal phase, H. These peaks are indicated by the dotted lines, and have been indexed by 
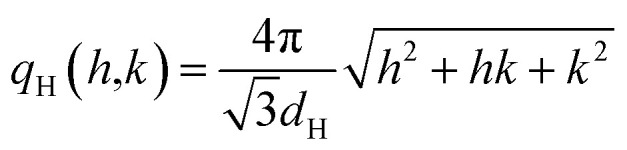
 where (*h*,*k*) are the (1,0), (1,1), (2,0) reflections and *d*_H_ = 3.0 nm. The fact that we do not observe peaks with the *l* component different from zero belonging to the hexagonal phase tells us that this phase is separated by the lamellar phase. The average size of this phase is about 38 nm, smaller than the lamellar phase.

We can calculate the electron density maps for these two phases. Both the lamellar and the hexagonal phases are centrosymmetric. In a centrosymmetric unit cell, the electron density, *ρ*(*r*) as function of position *r*, can be written as a Fourier series of cosines given by1
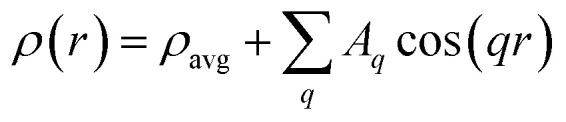
where *A*_*q*_ are the Fourier coefficients, *q* the Fourier vector, and *ρ*_avg_ is the average electron density. In the small angle range, the Lorentz correction can be obtained by dividing the integrated intensities of the diffraction orders by the magnitude of the reciprocal lattice vector.^54^ The peak intensity has been divided by the multiplicity of the reflection. Finally, the relative phases of the diffracted orders have been chosen in agreement with Turner^55^ for the hexagonal phase, +−−, and a with the +++−−−− sequence for the lamellar phase,^50^ to get a profile with a chemically consistent ratio of 1.4 between the core size and the chain length. In panel (c) and (d) we show the calculated electron density, *ρ*(*r*), for the supramolecular lamellar and hexagonal organization, respectively.

At lower *q*-values, *q* < 1.2, the SAXS profile, *I*(*q*), shows a power law behavior and a large peak around *q* = 0.6 nm^−1^ suggesting the formation of a network of correlated nanoregion defects^56,57^ occurring during the NCs assembly and aggregation into larger particles, P (see the gray area in panel (b)). In order to describe and quantify these nanoregion defects, we assume a theoretical model consisting of a volume fraction of interacting hard spheres with radius *R* and distance 2*R*_HS_. We find the fitted hard-sphere diameter *R*_HS_ is consistent with the size of the particles *R* = 5 nm, indicating a pure hard sphere model of defects (see ESI,† SAXS model for details). These correlated defect nanoregions permeate the lamellar and the hexagonal phases assembling into larger particles, P, with sharp interfaces, since the *P*_B_ exponent is found to be −4 indicating a Porod regime. The resulting structure is sketched in panel (e). The maximum size of each NC can be estimated by *ξ*_NC_ = *d*_L_ and *ξ*_NC_ = *d*_H_ in the lamellar and hexagonal phase, respectively, in agreement with the TEM measurements, while its atomic structure remains to be determined due to the difficulty of synthesizing single crystals suitable for an X-ray diffraction structure solution. For this aim, a crystal growth with slower kinetics is in progress; in addition to possible single crystals suitable for a structure solution, this also will allow us to get a system with a different degree of disorder and to study its effect on material functionality.^56,57^ Indeed, intriguing properties are found to be related to AgNCs supramolecular assembly. For example, the compression of Ag–Ag distance in different supramolecular aggregates, is responsible for lower emission energy^58^ and morphological transformation leads to multicolour light emissions^59^

Different supramolecular structures of our AgNCs can be obtained by changing some parameters such as the concentration of the ligand, the reaction temperature, and limited available reaction volume. We have preliminarily investigated the electronic conductivity of the Ag _38_(SC_2_H_4_Ph)_24_ nanocluster using C-AFM measurements. Nanoclusters have evidenced a strong anisotropy, *i.e.* the sample is highly conductive in the plane and non-conducting out of the plane. This property could find applications in sensor devices. Further detailed investigations on the AgNC growth, assembly and functionality are still in progress.

### 2.3 Modeling

In the absence of experimental structural information on the Ag_38_(SRN_3_)_24_ system, we resorted to a theoretical structure prediction protocol involving a stochastic search among thousands of possible candidate models. We therefore performed a DFT Global Optimization (GO) by using the Basin Hopping (BH) algorithm^60^ as implemented in an in-house python code. To identify the Global Minimum (GM), the algorithm starts from (1) an initial sample of 3*N* arbitrary atomic coordinates (*N* is the nuclearity of the investigated system), (2) generates a new configuration *via* a random perturbation of the coordinates and a subsequent local optimization, (3) accepts or rejects the new configuration according a Metropolis criterion (*i.e.* with probability min(1,exp(Δ*E*/*T*)), where Δ*E* is the energy difference between the initial and new configuration and *T* is the absolute temperature). The procedure iteratively continues with the random generation of new local minima starting from the current accepted one.

To accelerate the DFT Potential Energy Surface (PES) exploration to a computationally affordable effort, we simplified the Ag_38_(SRN_3_)_24_ system by replacing the RN_3_ moieties with H atoms. We therefore assumed that the energy ordering of the configurations generated from the BH algorithm is only slightly affected by the shape of the ligands that bind the S atoms. Additionally, we validated this assumption by verifying that the energy ordering of a few configurations, extracted from the GO procedure, was conserved when replacing the H atoms with bulkier methyl ligands.

We performed two different BH runs, hereafter marked as BH-A and BH-B, each of which generated about 2700 local minima. In the BH-A run, the GM search started from a structure derived from the chiral Au_38_(SC_2_H_4_Ph)_24_ nanocluster^27^ by replacing the Au with Ag atoms and the R groups with H atoms. The initial configuration (Ini-A) is symmetric like the parent and possesses a *D*_3h_ symmetric Ag_23_ core with six dimeric Ag_2_S_3_ staples and three short monomeric AgS_2_ staples organized according to a *D*_3_ symmetry around the Ag_23_ core. In the BH-B run we instead started the algorithm with a random configuration of the system Ag_38_(SH)_24_ (Ini-B).

**Fig. 5:**
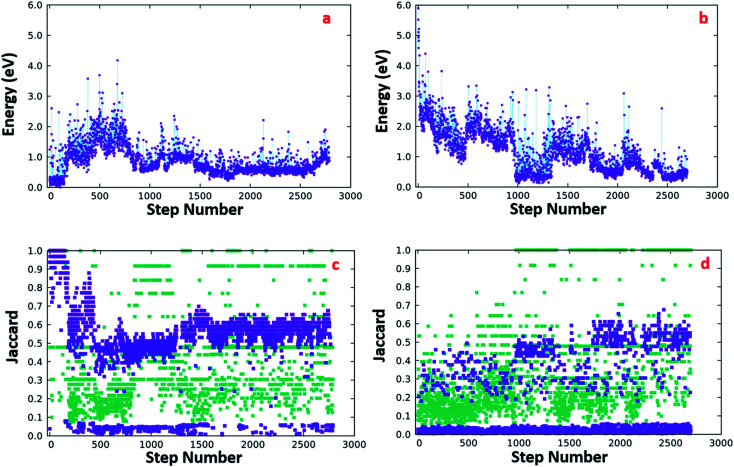
(Top-left) DFT energy as a function of the iteration number of the BH run-A whose initial structure was derived from the Q-Au_38_(SC_2_H_4_Ph)_24_ geometry.^61,62^ (Top-right) DFT energies of the structures generated by the BH run-B, whose starting point was a random Ag_38_(SH)_24_ structure. The energy reference for the data in panels (a) and (b) is the energy of the most stable structure we found through the structural search (both over the run A and B). (Panel c) Violet squares are Jaccard similarity coefficients measured by comparing the Ag_38_S_24_ fragment of each structure generated by the run-A with the Ag_38_S_24_ fragment of the lowest energy structure obtained from the same run; green squares are the Jaccard indices calculated by comparing the Ag_*n*_ core of each structure with the symmetric Ag_23_ core of the initial structure. (Panel d) Jaccard indices evaluated for the structures created by the GO algorithm over the run-B: the target structures were the same employed for the measure of the similarity coefficients depicted in panel (c).

Panel (a) in Fig. 5 reports the energy as a function of the iteration number for the BH-A run. We took as a reference the lowest energy among the local minima generated from the simulation, which coincides with the lowest-energy or putative global minimum (GM) of both BH-A and BH-B runs. The algorithm finds the most stable structure within the first 100 iterations; then, it escapes from the initial energy funnel to reach, around iteration 1800, a different local minimum, which is only 0.2 eV less stable than the previous one. Panel (c) of Fig. 5 reports the results of the similarity analysis we performed to compare the geometries generated from BH-A with the GM structure. Structural similarity is measured using Jaccard indices,^63–65^ evaluated by employing the heuristic algorithms described in ref. 66 The violet squares are the Jaccard coefficients obtained from the comparison of structures without taking into account the H atoms. The green squares are, instead, Jaccard coefficients obtained when comparing the structures' metallic core. Here, we define the metallic core as the complementary subset to the Ag atoms whose nearest neighbors are two S atoms. By inspecting the data reported in panel (c) of Fig. 5, we find that the GM's Ag_38_S_24_ fragment retains the symmetry of the initial Ag_38_(SH)_24_ structure, differing only in a more stable arrangement of the H atoms. Away from this initial energy-funnel, the local minima visited by the algorithm are all less stable than the GM due to either or both the following reasons: (1) a distortion of the metallic core and/or (2) a different organization of the AgS units around the metallic core.

Considering the BH-B run, panel (b) shows that our algorithm, even starting from a random Ag_38_(SH)_24_ configuration, is able to find, in a few hundred iterations, isomers at very low energy, less stable than the GM by only about 0.1 eV. Despite the presence of configurations whose energy differs by a few tenths of an eV from the GM energy, the similarity analysis reported in panel (d) (Fig. 5), indicates that none of the configurations generated from BH-B is structurally identical to the GM. However, a deeper analysis shows that the lowest energy structure (LES-B), generated from the BH-B run, has an Ag_23_ core that is isomorphic to the GM's one, and the remaining Ag_15_S_24_ shell is constituted by 6 dimeric Ag_2_S_3_ and 3 monomeric AgS_2_ units, exactly as in the GM structure. Thus, LES-B differs from the GM only in a slightly less symmetric arrangement of the staples. All in all, the above stochastic sampling strongly supports the assignment of the GM structure.

Beside the geometrical structure, we also simulated the absorption spectrum of the two lowest energy isomers generated from the GO by performing time-dependent DFT (TDDFT) simulations. Before calculating the optical spectra, we replaced the H with CH_3_ ligands to better take into account the effect of organic aliphatic ligands (as the ones experimentally employed to isolate the Ag_38_(SRN_3_)_24_ compound) on the optical response of the NCs.^67^ To minimize the conformational energies of the added CH_3_, ligands we followed a three-step procedure that involves the initial relaxation of the ligands, a constrained MD lasting 10 ps at 600 K during which the AgS core was kept frozen, and a final unconstrained relaxation of the last geometry generated from the dynamics. Due to the larger steric hindrance of methyl ligands, the energy difference of the structures generated from GM and LES-B increases by 0.2 eV with respect to the parent isomers.

**Fig. 6:**
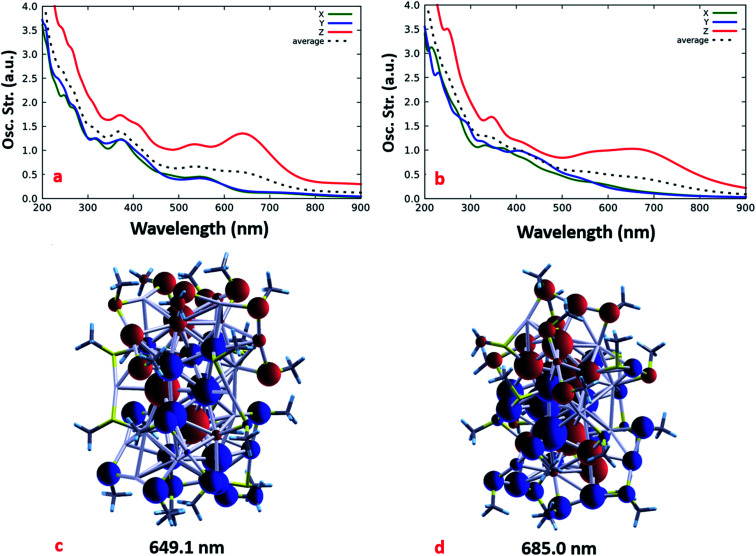
Simulated B3LYP-TDDFT average absorption spectra (dotted line) and their decomposition into Cartesian components for: the Global Minimum (GM, top-left) and the lowest energy structure obtained from BH-B (LES-B, top-right). Induced Mulliken charges are shown in the bottom-left for the GM calculated under irradiation with 649.1 nm light, and are shown for LES-B in the bottom-right calculated under irradiation with 685.0 nm light. Positive IMC are represented with in red.

Fig. 6 contains the calculated electronic spectra of the structures derived from GM and LES-B, whose skeletons are visible at the bottom of the figure. Both of them have an oblate shape whose geometric anisotropy, reflected in the polarizability, leads to a larger optical response when the light is polarized along the long axis of the structure. The limited extension of the NC in the plane perpendicular to the long axis limits the electronic polarizability, and reduces the average optical absorption in the visible range, in tune with the measured UV spectrum. The bottom panels in Fig. 6 show, for both the isomers, the Induced Mulliken Charges (IMC) corresponding to the lowest energy excitation. Simulations indicate a charge separation, characteristic of incipient plasmon excitations, which is known to depend on the morphology of the metal core.^68^ Being more pronounced in the isomer derived from the GM, the charge separation gives an Oscillator Strength (OS) bigger than in the LES-B structure containing a less symmetric Ag_38_S_24_ fragment. This is especially evident in the low energy part of the spectra.

Considering the proximity in energy of the putative GM and LES-B structures and that the asymmetric conformations of the Ag_38_S_24_ fragment are entropically favored, we expect that such conformations will also be populated by the system in its dynamics at room temperature, and we therefore predict a smoothing of the actual absorption spectrum with respect to the well-defined peaks of the GM structure. This smoothing should be further enhanced by vibrational broadening (note that the calculated vibrational broadening, introduced by artificially damping the calculated dipole signal, is underestimated), thus being more in tune with and accounting for the weaker features of the experimental optical absorption spectrum. It should be underlined however that, despite the broadening, both the putative GM structure and the LES-B exhibit a pronounced anisotropy, which we therefore predict will not be smoothed by the system's dynamics. Plausibly the anisotropy could also affect other properties such as conductivity.

As the third step in our modeling protocol, we employed the annealing technique starting from the best Ag_38_S_24_ model generated from the GO to investigate how the ligands' conformation affects both the energy and geometry of interacting NCs. We considered two different isomers for the annealing. The former, derived from the GM after replacing the H atoms with the RN_3_ moieties (called hereafter folded-isomer), has a compact structure with ligands folded around the metal core. The folding was obtained by shaking the RN_3_ groups in 5 ps DFT-AIMD at 600 K and then relaxing the resulting structure. The latter isomer (unfolded-isomer), whose RN_3_ ligands are unfolded, has been generated by relaxing at a classical level, the ligands of the folded structure in the presence of a positive charge of 5*e*-on each terminal N_3_ group: this extreme structure has been generated only to obtain a starting geometry where the RN_3_ ligands are totally unfolded but correctly bonded to the metal/sulfur shell.

When applied to the folded-isomer, the classical-FF annealing procedure returns a structure similar to the compact, folded structure obtained by *ab initio*: the gyration radius *R*_g_ of the DFT nanocluster is in agreement with that of the annealing/minimization procedure: *R*_g_ = 10.114 Å for the DFT structure, and *R*_g_ = 11.46 Å for the MD structure. When considering the unfolded isomer, the annealing produces a structure in which the backbones of the ligands are almost unfolded, with a prevalence of anti-configuration in the dihedral angles of the alkyl chain. The size of the unfolded nanocluster is much bigger than that of the folded nanocluster: the volume of the unit cell in which the nanocluster can be enclosed is 38.4 nm^3^ and 30.9 nm^3^ for the unfolded and folded structures, respectively. From an energy point of view, this different topology results in about 20 kcal mol^−1^ of energy gain for the folded nanocluster with respect to the unfolded one. This can be explained in term of stronger van der Waals interactions between adjacent carbon chains in the compact nanocluster, whereas in the unfolded structure the carbon backbones of the ligands are much further apart and the dispersive interactions smaller.

**Fig. 7:**
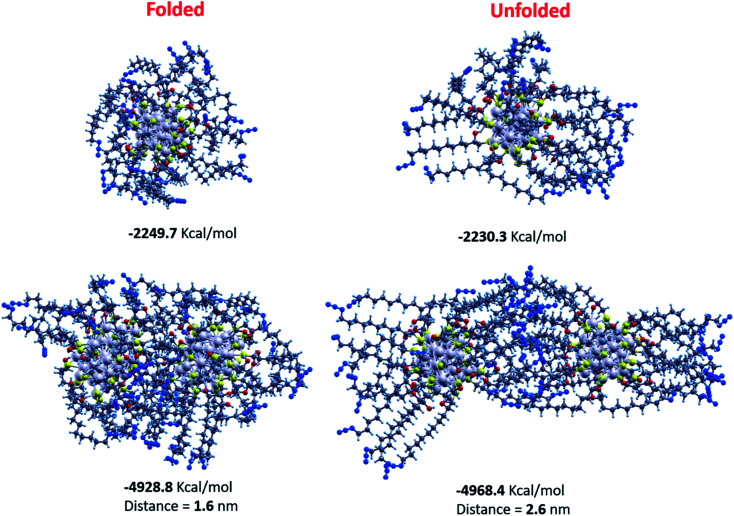
Graphical representation of the nanocluster structures calculated by the annealing/minimization procedure. Top left panel reports the single nanocluster geometry obtained by starting with the DFT geometry. In the top right panel is shown the geometry calculated by starting with the unfolded nanocluster structure. The bottom left panel represents the interaction between two folded nanoclusters, whereas in the bottom right panel the picture is relative to the interaction between two unfolded nanoclusters. Ag atoms are indicated in light grey, sulphur atoms in yellow, oxygen atoms in red, carbon atoms in dark grey, hydrogen atoms in light blue, nitrogen atoms in blue.

To investigate the geometric and energetic properties playing a role during the self-assembly of the NCs, we investigated, as a prototype of such interactions, the forces acting between two NCs. These are very different in the case of the compact geometry with respect to that of the unfolded structure as shown in the bottom panels of Fig. 7. In particular the distance between the geometrical centers of the two NCs is 1.6 nm for the folded conformers, whereas it is almost doubled, 2.6 nm in the case of the unfolded topology, which is much closer to the XRD data of d*H* = 3.0 nm. The energetics also shows drastic differences in the two topologies: the binding energy between the two nanoclusters is now clearly in favor not of the folded structure but of the unfolded structure, by about 80 kcal mol^−1^. This striking inversion can be explained in terms of a much more efficient inter-digitation among the long alkyl chains and N_3_ groups in the case of the two unfolded nanoclusters, where the van der Waals energy is the main term that leads to such an energy-difference. This suggests that NC–NC contacts can be formed in an exponential number of possible different configurations, which can explain the experimental difficulty in producing good-quality single crystals and the variety of phases determined *via* SAXS and WAXS measurements discussed above.^69^

## 3. Conclusion

In summary, the fluorescent Ag_38_(11-azido-2-ol-undecane-thiolate)_24_ NC here prepared and thoroughly characterized *via* spectroscopic and computational methods, presents a unique combination of attractive features, both from a scientific perspective and in view of technological applications. First, it possesses an atomically precise stoichiometry and structure, here demonstrated *via* computational tools and by analogy with similar systems, while single crystal X-ray diffraction to confirm theoretical predictions is in progress. Second, it exhibits fluorescence with two different emission signals, in the visible and in the NIR region centered at 630 nm, especially useful in biomedicine for imaging NCs where the tissue absorption of light is very low. Third, the synthesis developed here is easy, economic, and environmentally benign, employing sodium ascorbate as a “green” reducer and foremost, its use as a mild reducing agent allows us to introduce and keep intact a terminal azido group in the thiolate (to the best of our knowledge for the first time in the field of MPC species). By way of “click” chemistry it is possible to easily and stably bind molecules with a triple C≡C bond using the NC as “molecule-carrier” for applications in several different fields (catalysis, sensors, imaging, biomedicine, *etc.*.). Preliminary results as catalyst support of a chiral β-amino alcohol ligand in asymmetric catalysis are comparable with the ones obtained in the homogeneous phase in terms of product yield and enantiomer selectivity showing, in addition, easy catalyst recovery and recycling.

Moreover, X-ray scattering data show that the NCs form disordered crystalline domains with different orientations self-assembling at nanoscale in lamellar and hexagonal superstructures permeated by nanoregion defects. The disorder found on both the atomic and nanometer scales makes difficult the growth of a single crystal that could provide the atomic structure of the NC. We have thus used theoretical modeling to gain insight into the structure and possible interactions of NCs. An extensive computational structural search confirms the cluster thermodynamic stability and predicts an atomistic structure of the Ag_38_S_24_ NC homologous to its Au analogue, the well-known Au_38_S_24_ NC. The simulated absorption spectrum is also in fair agreement with the UV-vis experimentally measured one. Finally, MD simulations of NC–NC interactions suggest a strong preference at the solid state for thiolate “brush” inter-digitized configurations over folded ones.

## 4. Experimental section

### Materials

All the chemicals were commercially available and were used without purification. AgNO_3_ (99%), Na_2_S_2_O_3_·5H_2_O (99.5%), ethanol (96%), acetone and dichloromethane were purchased from Sigma Aldrich. Sodium ascorbate and Water PLUS for HPLC were acquired from Carlo Erba.

### Synthesis of the Ag_38_(11-azido 2-ol-undecane-thiolate)_24_ nanocluster[Ag_38_(SRN_3_)_24_]

The procedure consists of five steps for the preparation of the azido ligand (reported in ESI†) and two steps for the preparation of the AgNCs. Briefly, first the ligand 11-azido-1-bromoundecan-2-ol is prepared. Then bromide is substituted for the thiosulphate group (SSO_3_) and the ligand is reacted with AgNO_3_. The S–SO_3_ bond easily cleaves forming the polymeric thiolate complex [(Ag^+^)_*x*_(SRN_3_)_*y*_]. It is then reacted with sodium ascorbate to partially reduce Ag^+^ ions to Ag^0^ atoms forming the nanocluster core whereas Ag^+^ thiolate groups build the protective shell around the core.

### Synthesis of sodium 11-azido-1-thiosulphate undecyl-2-ol

The procedure is reported in our previous publication.^5^ To avoid the unpleasant thiol smell, we first prepared the N_3_R-thiosulphate sodium salt (N_3_RS–SO_3_Na^+^) by reacting 11-azido-1-bromoundecan-2-ol N_3_R–Br (R = C_11_H_22_O) with sodium thiosulphate pentahydrate (Na_2_SSO_3_·5H_2_O). 900 mg (3.08 mmol) of N_3_R–Br were dissolved in 15 mL ethanol. 3.06 g (12.32 mmol) Na_2_SSO_3_·5H_2_O dissolved in 10 mL H_2_O were added to the N_3_R–Br solution and heated to reflux under magnetic stirring overnight. The solvent was evaporated using a Rota vapor, the residue purified by washing with hot water and ethanol, well dried under vacuum and finally extracted twice with hot acetone. Yield: 91%.

### Synthesis of Ag^+^11-azido-2-ol-undecanethiolate polymeric complex (Ag^+^)_*x*_(SRN_3_)_*y*_

437 mg (1.26 mmol N_3_RSSO_3_Na) is dissolved in 6 mL ethanol and, under magnetic stirring, a solution of 214 mg (1.26 mmol) AgNO_3_ in 20 mL H_2_O is added. After 5 minutes, the solution turns opaque and a white/yellowish colloidal suspension is formed. After 3 hours stirring at 30 °C, the product is separated by centrifugation, washed thoroughly with H_2_O, ethanol, extracted more times with acetone and finally the pale yellow precipitate is dried under vacuum.

### Synthesis of Ag_38_ (11-azido, 2-ol undecanethiolate)_24_ nanocluster [Ag_38_(SRN_3_)_24_]

150 mg of (Ag^+^)_*x*_(SRN_3_)_*y*_ polymeric complex are dispersed in 120 mL of ethanol. 600 mg (3.40 mmol) of sodium ascorbate are dissolved in 10 mL of hot water and added to the (Ag^+^)_*x*_(SRN_3_)_*y*_ dispersion, then, after addition of 30 mL of dichloromethane, the mixture, under magnetic stirring, is warmed up to 80 °C for three hours and left at room temperature under magnetic stirring overnight. The dispersion is centrifuged at 3000 rpm for 15 minutes and the red brown precipitate is extracted more times with dichloromethane. All the yellow brown dichloromethane fractions so obtained are then evaporated in a Rota vapor and the red brown residue dried under vacuum. Yield: 62% referred to AgNO_3_. Elemental C, H, N, S, analyses (w/w%): C = 32.10, H = 5.41, N = 9.75, S = 7.83. Theoretical for Ag_38_(SRN_3_)_24_: C = 31.82, H = 5.34, N = 10.12 S = 7.72.

### Instruments

Morphologic investigation was performed using a JEOL JEM 2010 Transmission Electron Microscopy (TEM). A few drops of the sample dispersion in hexane were deposited on a Cu grid and the solvent was let to evaporate at room temperature. The composition of the samples was investigated by way of Elemental C, H, N, S analyses measurements, performed using a Carlo Erba EA 1108 microanalyser at CNR-ISM. Attenuated Total Reflectance Fourier Transform Infrared (ATR-FTIR) spectra were recorded on a Shimadzu IRPrestige-21 to study structural features. Optical characterization was accomplished by way of UV-vis and fluorescence measurements. UV-visible spectral analyses were performed on a Lambda 950 Perkin Elmer spectrophotometer. Steady-state fluorescence spectra for AgNC solutions were recorded using a Fluorolog 3 (Horiba-JobinYvon) spectrofluorometer, with 5 nm grids for both excitation and emission. The emissions have been collected, in the range 410–780 nm, exciting the sample diluted in CH_2_Cl_2_ at 400 nm. The corresponding excitation spectra have been collected in the range 270–600 nm, under different excitation wavelengths between 500 nm and 650 nm. All experiments were performed at room temperature using quartz cuvettes with an optical path length of 10 mm. Quantum yield measurements have been performed, using Rhodamine 6G in ethanol (QY 0.96) as standard. 400 nm has been chosen as the excitation wavelength and the emission of Rhodamine 6G and AgNCs (at different concentrations) have been integrated from 500 to 780. The quantum yield of silver nanoclusters is obtained using the following equation^70^*φ*_AgNCs_ = φ_Rh_(*m*_AgNCs_/*m*_Rh_)(*η*_1_/*η*_2_)^2^ where *φ* is the quantum yield, *m* is the slope of the gradient of the plot of integrated fluorescence intensity against absorbance, *η* is the refractive index of the solvents, 1 for ethanol and 2 for CH_2_Cl_2_, respectively.

Wide Angle X-ray Scattering patterns were acquired at the XRD1 beam-line of the Elettra Synchrotron facility in Trieste, Italy.^49^ The beam energy was set to 1 Å through a vertical collimating mirror and a double-crystal Si(111) monochromator followed by a bendable focusing mirror and directed to the sample on the top of a glass fiber. The diffracted signal has been acquired with a 2D detector (Dectris Pilatus 2M) with 1475 × 1679 pixels of 172 × 172 μm^2^ area. The sample to detector distance was set to 99.62 mm. During the measurement, the sample was rotated 360° around the fiber axis. The LaB_6_ powder X-ray diffraction was used to calibrate the collected patterns.

The Small Angle X-ray Scattering (SAXS) measurements were performed with a Xeuss 2.0 Q-Xoom system (Xenocs SAS, Grenoble, France), equipped with a micro-focus GIenix 3D X-ray Cu source (*λ* = 1.54 Å), a two-dimensional Pilatus3 R 300K detector placed at variable distance from the sample and an additional Pilatus3 R 100K detector at a fixed shorter distance from the sample (around 14 cm) and tilted at 36 degrees to access larger scattering angles (Dectris Ltd., Baden, Switzerland). Calibration of the sample-detector distance was performed using silver behenate for the small-angle region and Al_2_O_3_ for the fixed-distance wide-angle detector. The solid samples were loaded into 0.5 mm thick washers used as spacers, closed with sticky Kapton windows and placed in the instrument sample chamber at reduced pressure (∼0.2 mbar). The beam size was defined to be 0.5 mm × 0.5 mm. The “dark” counts were subtracted from the two-dimensional scattering patterns, and then masked, azimuthally averaged, and normalized for transmitted beam intensity, exposure time and subtended solid angle per pixel, by using the FoxTrot software developed at SOLEIL. The contributions of the empty polymeric windows were then subtracted from the one-dimensional *I vs. q* profiles (*q* = 4π sin(*θ*)/*λ*, where 2*θ* is the scattering angle). Data collected with the SAXS detector at 30, 100 and 200 cm from the sample, and with the WAXS detector were merged to obtain an overall scattering vector range of 0.07–32 nm^−1^. The reported (dimensionless) intensity values are absolute scale units (cm^−1^) multiplied by the effective sample thickness expressed in cm.

### Theoretical methodology

*In silico* simulations were performed for predicting the structure of the most stable isomer of the Ag_38_(SRN_3_)_24_ system, its optical properties, and the energetics and the optimal inter-cluster distance when they are packed closely together into a lattice structure. The size of the system does not allow a full *ab initio* exploration of the complex potential energy landscape that describes the dense manifold of conformers. Hence, we separated the structure generation problem into two parts and adopted a combined computational strategy in which (i) high-level *ab initio* methodologies are first used to explore and predict the morphology of the metal-sulfur Ag_38_S_24_ cluster single component, followed by (ii) a study of the interaction between the ligands performed by means of a classical force field, which is well parameterized for standard intermolecular interactions involving alkyl chains with chemical groups such as OH or N_3_, and allows us to investigate the nanocluster packing problem. The single-cluster structure generated from step (i) has also been used (iii) to predict the optical response of these systems, to be compared with experimental absorption spectra in solution as customary.^71^

These three steps then correspond to different simulation methods. We employed Monte Carlo Global Optimization (GO) techniques to unravel the atomic arrangement of the Ag_38_S_24_ fragment; using Time-Dependent Density-Functional Theory (TDDFT) simulated optical properties; and finally we resorted to classical molecular dynamics for studying, by annealing techniques, the organization of interacting Ag_38_(SRN_3_)_24_ clusters in the solid state.

Global optimization runs were conducted using an in-house python implementation of the Basin Hopping algorithm.^60^ The energies of the local minima were calculated at the DFT-PBE^72^ level by employing the OPENMX code [http://www.openmx-square.org/], which solves the Khon–Sham equations within the pseudo potential-LCAO framework by using localized pseudo-atomic numerical basis sets.^73^ The OPENMX code describes the orbitals as a linear combination of localized functions, and reduces the computational burden, related to the evaluation of the Hartree potential, with the aid of a fast FFT solver that needs the definition of a cut-off energy.^74^ In our simulations, the Kohn–Sham energies have been evaluated by providing OPENMX with DZVP basis sets, norm-conserving pseudopotentials, and an energy cutoff of 150 Ry [https://t-ozaki.issp.u-tokyo.ac.jp/vps_pao2019/]. The OPENMX code was also employed for performing Nose–Hoover *NVT* MD simulations^75^ on the two Ag(_38_SCH_3_)_24_ structures generated by starting from the two most stable local minima extracted from the GO procedure. A similar dynamics, lasting 5 ps was also performed on the structure generated from the GM after replacing the H atoms with the RN_3_ ligands.

TDDFT simulations were performed using the CP2K package [http://doi.org/10.1002/wcms.1159]^76,77^ that solves the Kohn–Sham equations with the mixed Gaussian and Plane Wave approach (GPW) proposed in ref. 78 and 79 The code's efficiency, already improved due to the exploitation of auxiliary plane-wave basis sets, takes further advantage of the use of pseudopotentials for describing the core electrons. The electronic energies were evaluated by employing DVZP primary Gaussian basis sets,^80^ norm conserving GTH-pseudopotentials^81^ and an auxiliary plane-wave basis set with a cutoff of 300 Ry. We choose the real-time (RT) TDDFT approach^82^ and the hybrid B3LYP functional^83^ for the prediction of the spectroscopic properties of the investigated systems. The computational burden due to the evaluation of the Hartree–Fock exchange was reduced by employing the Auxiliary Density Matrix Method (ADMM).^84,85^ The optical response of the clusters was obtained by following the evolutions of the system's electric dipole after perturbing the equilibrium state by electric fields with a strength of 0.0005 a.u polarized along different Cartesian directions. The dipole-dynamics, lasting 9 femto-second, were sampled with time steps of 0.012 femto-second. A time damping of 7.2 fs (corresponding to an FWHM of 0.25 eV) was chosen to broaden the simulated spectrum.

To identify the most stable arrangement of pairs of interacting Ag_38_(SRN_3_)_24_ clusters, we employed the Tinker software^86^ to perform an annealing procedure based on classical force fields. The force field used in the calculations is the Allinger MM3 (ref. 87) with the addition of interaction terms due to the Ag–S bond, S–Ag–S, Ag–S–Ag, Ag–S–C angles, and the dihedral angle Ag–S–C–C. All of these energy terms have been determined by fitting the potential energy curve determined by *ab initio* DFT calculations on the Ag_2_(SCH_3_)_2_ molecule (ESI, Fig. S1†). DFT energies, reported in the ESI, Fig. S5–S9,† were calculated by using the Gaussian code with the B3LYP functional and 6-31++g** basis set.^88–94^ The parameters for the N_3_ group have been taken from data in the literature.^95^ The topology of the atoms employed in the annealing calculations is reported in the ESI Table S1† based on the molecular structure of the ligands identified by Bertorelle,^96^ which are reported in the ESI, Fig. S1–S3,† where CH_3_ is used as the alkyl group. Similar fragments have been identified in the most stable geometries generated from the DFT-GO, which contain three L1 ligands (R–S–Ag–S–R) and six L2 ligands (R–S–Ag–S(R)–Ag–S–R) arranged around the Ag_23_ core (ESI Fig. S4†).

Thus, to describe with classical forces the Ag_38_(SRN_3_)_24_ structure we defined two S atom types: the trivalent and the bivalent. The former binds two Ag atoms and one carbon atom (atom identifier 17 in the ESI Table S1†), whereas the latter binds one Ag atom and one carbon atom (atom identifier 15 in the ESI Table S1†). Silver atoms have, instead, been classified in three different groups: core atoms (atomic id: 152), Ag belonging to ligands and bound to two core atoms (atomic id: 158), and Ag belonging to ligands and bound to three core atoms (atomic id: 157). The other energy terms used in the annealing have been taken by the MM3 force field and are reported in the ESI Tables S2–S8.†

The annealing procedure, starting at a temperature of 300 K, involves an initial equilibration lasting 10 ps, followed by 90 ps of cooling dynamics during which the temperature linearly decreases to zero. The time step employed for integrating the equations of motion was 1.0 fs.

The resulting geometry of the annealing of the single nanocluster has been optimized by limited memory L-BFGS minimization over Cartesian coordinates using a modified version of the algorithm of Jorge Nocedal and with the RMS Gradient threshold per atom fixed at 0.05 kcal mol^−1^ Å^−2^.

## Author contributions

The manuscript was written through contributions of all authors. All authors have given approval to the final version of the manuscript. Gaetano Campi, Lorenza Suber and Luca Sementa contributed equally.

## Conflicts of interest

The authors declare no competing financial interest.

## Supplementary Material

NA-003-D1NA00090J-s001
