# Supplementary material for: Design of a fluorescent and clickable Ag38(SRN3)24 nanocluster platform: synthesis, modeling and self-assembling
Source: Nanoscale Adv. 2021 Apr 7;3(10):2948–60. doi: 10.1039/d1na00090j (PMC9418538; doi:10.1039/d1na00090j)
Supplement: NA-003-D1NA00090J-s001 [file NA-003-D1NA00090J-s001.pdf]

Electronic Supplementary Material (ESI) for Nanoscale Advances.
This journal is © The Royal Society of Chemistry 2021

Supporting Information

Materials. All the chemicals were commercially available and were used without purification. Tetrabromomethane, triphenylphosphine, dichloromethane, acetone, water for HPLC, DMSO dry, sodium azide, lithium bromide and Amberlyst 15 were purchased from Sigma Aldrich.

Instruments. ^{1}H NMR spectra were recorded using a a Bruker Avance 400 (400 MHz). Residual solvent peaks were used as internal references for ^{1}H NMR spectra: chloroform (δ 7.26 ppm). Chemical shifts (δ) are reported in parts per million (ppm) and coupling constants (J) are reported in Hz. Splitting patterns are designated as s, singlet; bs, broad singlet; d, doublet; bd, broad doublet dd, doublet of doublets; ddd, doublet of doublet of doublets; t, triplet; bt, broad triplet; dt, doublet of triplets; ddt, doublet of doublet of triplets; q, quartet; m, multiplet. ^{13}C spectra were recorded using a or a Bruker Avance 400 (101 MHz). Chemical shifts (δ) are reported in parts per million (ppm) relative to the internal standard of the residue solvent peak: chloroform (77.2 ppm).

The preparation of the ligand 11-azido-1-bromoundecan-2-ol is accomplished as sketched in the following synthetic scheme:

Synthetic scheme:

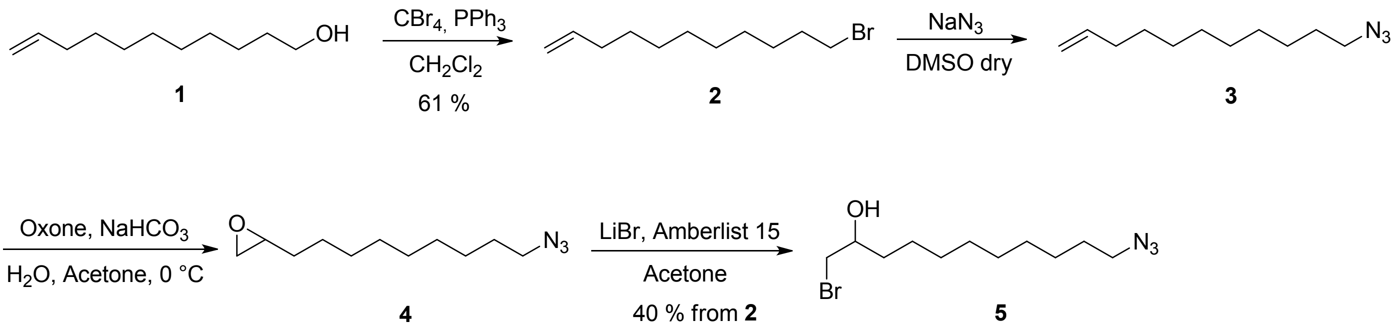

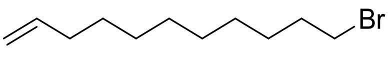

11-bromoundec-1-ene (2): 1.25 mmol of CBr_4 (420 mg) were added to a solution of **1** (1 mmol, 0.20 ml) in 2.5 ml of CH_2Cl_2. The mixture was cooled at 0 °C and 1.88 mmol (490 mg) of PPh_3 were added in portions. The reaction mixture was stirred at room temperature for 12 hours and the solvent was evaporated *in vacuo*. The residue was dissolved in diethyl ether and the insoluble white solid eliminated by filtration. This operation was repeated several times until the complete removal of the solid. The crude product was purified by flash chromatography (Hex/AcOEt, 96:4) to afford 142 mg of **2** (61 %). ^{1}H NMR (400 MHz, $CDCl_3$): δ 5.81 (ddt, J = 16.9, 10.2, 6.7 Hz, 1H, $C\underline{H}=CH_2$), 5.03-4.90 (m, 2H, $CH=C\underline{H_2}$), 3.40 (t, J = 6.9 Hz, 2H, $C\underline{H_2}Br$), 2.10-1.99 (m, 2H, $C\underline{H_2}CH=CH_2$), 1.91-1.80 (m, 2H, $C\underline{H_2}CH_2Br$), 1.48-1.23 (m, 12H, $=CHCH_2(C\underline{H_2})_6CH_2CH_2Br$). ^{13}C NMR (101 MHz, $CDCl_3$): δ 139.3, 114.3, 34.1, 33.9, 33.0, 29.5, 29.5, 29.2, 29.0, 28.9, 28.3.

11-azidoundec-1-ene (3): To a solution of **2** (1 mmol) in 1.3 ml of DMSO dry were added 4 mmol (260 mg) of NaN_3 under argon atmosphere. The reaction mixture was stirred at room temperature for 12 hours, then diluted with Et_2O and the organic layer was washed seven times with small portions of water. The aqueous layer was extracted with Et_2O three times, then the combined organic layers were washed with brine and dried over Na_2SO_4. The solvent was evaporated *in vacuo* to afford crude azide **3**. ^{1}H NMR (400 MHz, $CDCl_3$): δ 5.80 (ddt, J = 16.9, 10.2, 6.7 Hz, 1H, $C\underline{H}{=}CH_2$), 5.03-4.88 (m, 2H, $CH{=}C\underline{H_2}$), 3.24 (t, J = 7.0 Hz, 2H, $C\underline{H_2}N_3$), 2.09-1.99 (m, 2H, $C\underline{H_2}CH{=}CH_2$), 1.64-1.53 (m, 2H, $C\underline{H_2}CH_2N_3$), 1.42-1.26 (m, 12H, $=CHCH_2(C\underline{H_2})_6CH_2CH_2N_3$). ^{13}C NMR (101 MHz, $CDCl_3$): δ 139.2, 114.2, 51.6, 33.9, 29.5, 29.5, 29.2, 29.2, 29.0, 28.9, 26.8.

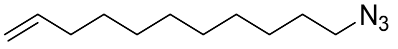

2-(9-azidononyl)oxirane (4): To a solution of crude azide **3** (1 mmol) in 15 ml of acetone were added 7.7 mmol (650 mg) of $NaHCO_3$ dissolved in 3.3 ml of water. The mixture was cooled at 0 °C and 5 mmol of Oxone (1.56 g) dissolved in 6.6 ml of water were added dropwise. The reaction mixture was stirred at room temperature for 2 hours, then it was filtered and concentrated *in vacuo*. The residue was dissolved in Et_2O and washed with water. The aqueous layer was extracted with Et_2O three times. The combined organic layers were washed with saturated $NaHCO_3$, brine, dried over Na_2SO_4 and the solvent was removed *in vacuo* affording the crude epoxy azide **4**. ^{1}H NMR (400 MHz, $CDCl_3$): δ 3.22 (t, J = 7.0 Hz, 2H, CH_2N_3), 2.92-2.83 (m, 1H, $OC\underline{H}CH_2$), 2.71 (dd, J = 5.0, 4.0 Hz, 1H, $C\underline{H_\alpha}H_\beta O$), 2.43 (dd, J = 5.1, 2.7 Hz, 1H, $CH_\alpha\underline{H_\beta}O$), 1.63-1.22 (m, 16H, $CH(C\underline{H_2})_8CH_2N_3$). ^{13}C NMR (101 MHz, $CDCl_3$): δ 52.4, 51.5, 47.1, 32.5, 29.5, 29.4, 29.4, 29.2, 28.9, 26.7, 26.0.

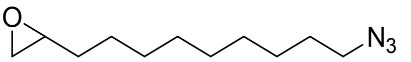

11-azido-1-bromoundecan-2-ol (5): To a solution of crude epoxy azide **4** (1 mmol) in 20 ml of acetone were added 4 mmol (0.35 g) of LiBr and 1 mmol of Amberlyst 15 at 0 °C. The reaction mixture was stirred at room temperature for 12 hours, then filtered through a pad of celite with acetone and concentrated *in vacuo*. The residue was dissolved in AcOEt and washed with water. The aqueous layer was extracted three times with AcOEt. The combined organic layers were washed with brine, dried over Na_2SO_4 and the solvent was evaporated *in vacuo*. The crude product was purified by flash chromatography (Hex/ AcOEt 8:2) to afford 201 mg of **5** (40 % over three steps). ^{1}H NMR (400 MHz, $CDCl_3$): δ 3.82-3.71 (m, 1H, $C\underline{H}OH$), 3.53 (dd, J = 10.3, 3.3 Hz, 1H, $C\underline{H_\alpha}H_\beta Br$), 3.37 (dd, J = 10.3, 7.1 Hz, 1H, $CH_\alpha\underline{H_\beta}Br$), 3.24 (t, J = 6.9 Hz, 2H, $C\underline{H_2}N_3$), 2.08 (bs, 1H, $O\underline{H}$), 1.65-1.26 (m, 16H, $CH(C\underline{H_2})_8CH_2N_3$). ^{13}C NMR (101 MHz, $CDCl_3$): δ 71.2, 51.6, 40.8, 35.2, 29.53, 29.5, 29.2, 28.9, 26.8, 25.7.

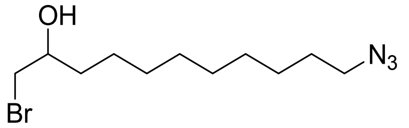

SAXS model

SAXS profile, *I(q)*, presents different features; alongside the main 3D lamellar phase and the 2D hexagonal phase described in the text (Fig. 4) we focus here on the low-q part (q<1.2 nm^{-1}) shown in the grey region of Fig. 4b. This part has been modelled by correlated defects in the hard sphere model, by the sum of two components:

$$I(q) = I_B(q) + I_D(q) \quad (1)$$

The first component, $I_B(q)$, predominating at lower q is given by a power law behavior with exponent P_B and it is ascribed to the formation of the large particles, P; the second one, $I_D(q)$, describes correlated defects nanoregions as interacting hard-spheres. The two components can be written as

$$I_B(q) = A_B + C_B q^{P_B} \quad (2)$$

$$I_D(q) = C_D S(q, R_{HS}, f_p) F(q,R) = C_D S(q, R_{HS}, f_p) \int_0^{\infty} P(R) \left[V(R)\Phi(R)R)\right]^2 dR \quad (3)$$

A_B and C_B are constant values and P_B is the power exponent. The $I_D(q)$ term takes into account the formation of spherical correlated defects with radius R; C_D is a constant independent of q and R; $V(R)$ and $\Phi(q,R)$ are the volume and the form factor of the single spherical particle, respectively. In order to account for the polydispersivity, σ, of the defect size, the intensity has been integrated over a Schultz-Zimm distribution of R, $P(R)$.64 Finally, the structure factor $S(q,R_{HS},\eta)$ has been modeled in the monodisperse approximation of hard spheres with radius R_{HS} and volume fraction η calculated with the Percus-Yevick equation. 65 We used the trust-region-reflective algorithm, 66 in order to fit the data and estimate the A_B and C_B constants, the power exponent P_B of the background term in $I_B(q)$, the constant C_D, the mean radius R and the dispersivity σ of the Schultz-Zimm defects distribution size $P(R)$, the hard sphere radius R_{HS} and volume fraction η in the Percus-Yevick approximation. We find $R=R_{HS}$=5 nm indicating a pure hard sphere defect distribution made of packed defects of 5 nm and a volume fraction of η=0.3.

This defect network, sketched in Figure 4e, permeates the lamellar and the hexagonal phases aggregating in larger particles, P, with sharp interface since the P_B exponent is found to be -4 indicating a Porod regime.

Table S1: Topology for the force field.

Atom id	Atom	Atom topology	Valence
10	N	Azide Terminal	1
15	S	S Bivalent	2
17	S	S Trivalent	3
45	N	Azide Central	2
107	N	Azide carbon-side	2
152	Ag	Ag Metal Core	0
157	Ag	Ag Ligand: bonds with 3 Ag-core atoms	2
158	Ag	Ag Ligand: bonds with 2 Ag-core atoms	2

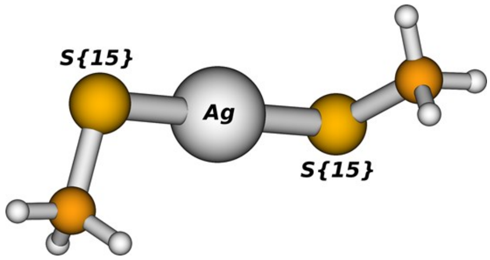

Figure S1: Structure of the L_1 ligand with the methyl as the alkyl chain. The atom identifiers of the sulfur are described in the main text.

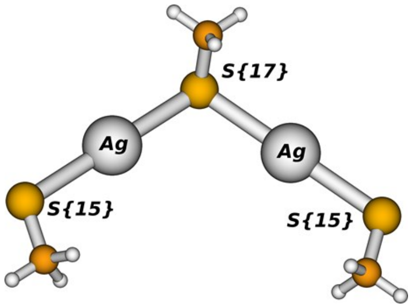

Figure S2: Structure of the L_2 ligand with the methyl as the alkyl chain. The atom identifiers of the sulfur are described in the main text.

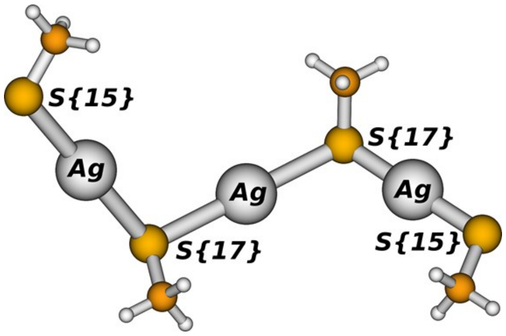

Figure S3 Structure of the L_3 ligand with the methyl as the alkyl chain. The atom identifiers of the sulfur are described in the main text.

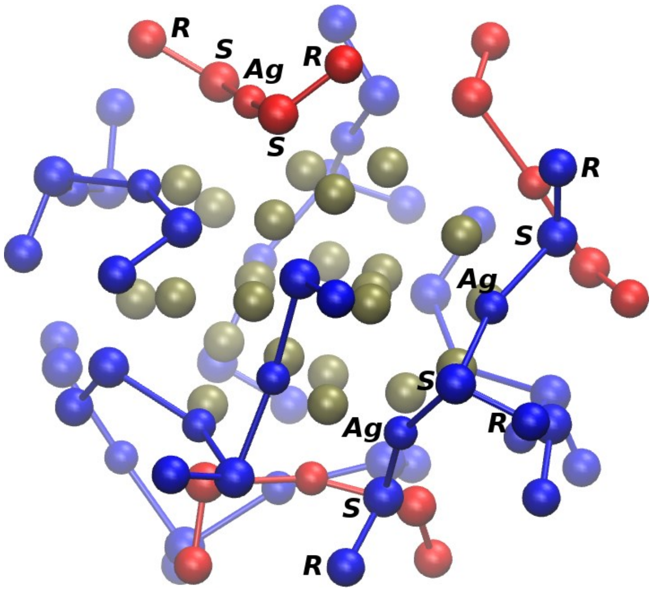

Figure S4: Representation of the nanocluster geometry calculated by DFT approach. In red are reported the backbone of the 3 L_1 ligands, in blue are the 6 L_2 ligands, and in tan are represented the 23 Ag atoms of the metal core. For sake of simplicity the labels are reported only for one L_1 and for one L_2 ligands.

Table S2: Van der Waals Interactions

Atom id	R (Å)	E (Kcal/mol)
15	2.15	0.202
17	2.15	0.202
45	1.93	0.043
107	1.93	0.043
152	2.5	0.200

Table S3:Van der Waals Pair Interactions

Pair Atom id		R (Å)	E (Kcal/mol)
152	15	2.7	1.10
152	157	2.75	37.65
152	158	2.75	37.65

Table S4: Hydrogen Bond Interactions

Pair Atom id		R (Å)	E (Kcal/mol)
15	21	2.68	1.35
17	21	2.55	0.60

Table S5: Bond Interactions

Pair Atom id		R (Å)	E (Kcal/mol/Å^2)
1	15	3.00	129.8517
1	17	2.95	129.4920
1	107	4.48	104.5288
10	45	18.6	82.0116
15	15	2.62	145.2469
15	157	1.81	172.6560
15	158	1.81	172.6560
17	157	1.81	172.6560

17	158	1.81	172.6560
45	107	11.30	88.4862

Table S6: Angle Interactions

Triplet Atom id			Force constant (kcal/mole/radian^2)	Θ^0 (deg.)	Θ^1 (deg.)	Θ^2 (deg.)
1	1	15	33.77	108.0	109.5	110.1
1	1	17	29.66	107.5	104.7	106.0
1	1	107	22.82	108.5		
1	15	1	38.33	95.9		
1	15	157	56.97	105.8		
1	15	158	56.97	105.8		
1	17	157	56.97	105.8		
1	17	158	56.97	105.8		
1	107	45	54.76	105.2		
5	1	15	33.77	110.8	110.8	108.0
5	1	17	32.63	105.0	105.0	107.7
5	1	107	38.10	108.5		
10	45	107	33.77	180.0		
15	157	15	33.36	165.7		
15	157	17	33.36	165.7		
15	158	15	33.36	135.7		
15	158	17	33.36	135.7		
17	157	17	33.36	165.7		
17	158	17	33.36	135.7		
157	17	157	10.97	100.0		
157	17	158	10.97	100.0		
158	17	158	10.97	100.0		

*(*the central atom of the angle is attached to 0, 1 or 2 additional hydrogen atoms, respectively*)

Table S7: Torsional Interactions

Quartet Atom id				Periodicity=1		Periodicity=2		Periodicity=3	
				Amplitude	Phase offset (deg.)	Amplitude	Phase offset (deg.)	Amplitude	Phase offset (deg.)
1	1	1	15	0.0000	0.0000	0.2000	180.0000	0.4000	0.0000
1	1	1	17	1.5000	0.0000	-0.8000	180.0000	0.1000	0.0000
1	1	1	107	-1.0000	0.0000	0.0000	180.0000	-1.2000	0.0000
1	1	15	1	-0.4400	0.0000	-0.2600	180.0000	0.6000	0.0000
1	1	15	15	-0.2000	0.0000	0.0000	180.0000	0.0000	0.0000
1	1	15	44	-0.5000	0.0000	0.0000	180.0000	0.2670	0.0000
1	1	15	157	0.3965	-0.2570	0.5456	-0.3133	0.7386	0.3878
1	1	15	158	0.3965	-0.2570	0.5456	-0.3133	0.7386	0.3878
1	1	17	1	-0.8000	0.0000	0.0000	180.0000	1.2000	0.0000
1	1	17	5	-0.8000	0.0000	-0.7000	180.0000	0.1000	0.0000
1	1	17	157	0.3965	-0.2570	0.5456	-0.3133	0.7386	0.3878
1	1	17	157	0.1983	-0.2570	0.2728	-0.3133	0.3693	0.3878
1	1	17	158	0.1983	-0.2570	0.2728	-0.3133	0.3693	0.3878
1	1	107	45	0.0000	0.0000	0.0000	180.0000	-0.4000	0.0000
1	15	15	1	1.8500	0.0000	-7.5550	180.0000	2.3400	0.0000
1	15	157	15	0.0000	0.0000	0.0000	180.0000	0.0000	0.0000
1	15	157	17	0.0000	0.0000	0.0000	180.0000	0.0000	0.0000

1	15	158	15	0.0000	0.0000	0.0000	180.0000	0.0000	0.0000
1	15	158	17	0.0000	0.0000	0.0000	180.0000	0.0000	0.0000
1	17	157	15	0.0000	0.0000	0.0000	180.0000	0.0000	0.0000
1	17	157	17	0.0000	0.0000	0.0000	180.0000	0.0000	0.0000
1	17	158	15	0.0000	0.0000	0.0000	180.0000	0.0000	0.0000
1	17	158	17	0.0000	0.0000	0.0000	180.0000	0.0000	0.0000
5	1	1	15	0.0000	0.0000	0.0000	180.0000	0.5400	0.0000
5	1	1	17	0.0000	0.0000	0.0000	180.0000	0.1250	0.0000
5	1	1	107	0.0000	0.0000	0.0000	180.0000	-0.4000	0.0000
5	1	15	1	0.0000	0.0000	0.0000	180.0000	0.6600	0.0000
5	1	15	15	0.3000	0.0000	0.0000	180.0000	0.6000	0.0000
5	1	15	157	0.0000	0.0000	0.0000	180.0000	0.6865	0.0150
5	1	15	158	0.0000	0.0000	0.0000	180.0000	0.6865	0.0150
5	1	17	1	0.0000	0.0000	0.0000	180.0000	0.6000	0.0000
5	1	17	5	0.0000	0.0000	0.0000	180.0000	0.7000	0.0000
5	1	17	157	0.0000	0.0000	0.0000	180.0000	0.6865	0.0150
5	1	17	158	0.0000	0.0000	0.0000	180.0000	0.6865	0.0150
5	1	107	45	0.0000	0.0000	0.0000	180.0000	0.0000	0.0000
6	1	1	15	-0.7000	0.0000	0.8000	180.0000	-0.1000	0.0000
6	1	1	17	-0.8000	0.0000	-1.2000	180.0000	-0.7000	0.0000
10	45	107	1	0.0000	0.0000	0.0000	180.0000	0.0000	0.0000
15	1	1	15	1.2500	0.0000	-0.3000	180.0000	0.0000	0.0000
15	1	1	17	0.0000	0.0000	0.0000	180.0000	0.0000	0.0000
15	1	15	1	0.0000	0.0000	-0.9000	180.0000	0.3000	0.0000
15	1	15	15	0.2000	0.0000	0.0000	180.0000	0.1000	0.0000
15	1	17	1	0.0000	0.0000	0.0000	180.0000	0.0000	0.0000
17	1	1	17	0.0000	0.0000	0.0000	180.0000	0.1250	0.0000
17	1	15	1	0.0000	0.0000	0.0000	180.0000	0.0000	0.0000
17	1	17	1	0.0000	0.0000	0.0000	180.0000	0.0000	0.0000
157	15	157	15	0.0000	0.0000	0.0000	180.0000	0.0000	0.0000
157	15	157	17	0.0000	0.0000	0.0000	180.0000	0.0000	0.0000
157	15	158	15	0.0000	0.0000	0.0000	180.0000	0.0000	0.0000
157	15	158	17	0.0000	0.0000	0.0000	180.0000	0.0000	0.0000
157	17	157	15	0.0000	0.0000	0.0000	180.0000	0.0000	0.0000
157	17	157	17	0.0000	0.0000	0.0000	180.0000	0.0000	0.0000
157	17	158	15	0.0000	0.0000	0.0000	180.0000	0.0000	0.0000
157	17	158	17	0.0000	0.0000	0.0000	180.0000	0.0000	0.0000
158	15	157	15	0.0000	0.0000	0.0000	180.0000	0.0000	0.0000
158	15	157	17	0.0000	0.0000	0.0000	180.0000	0.0000	0.0000
158	15	158	15	0.0000	0.0000	0.0000	180.0000	0.0000	0.0000
158	15	158	17	0.0000	0.0000	0.0000	180.0000	0.0000	0.0000
158	17	157	15	0.0000	0.0000	0.0000	180.0000	0.0000	0.0000
158	17	157	17	0.0000	0.0000	0.0000	180.0000	0.0000	0.0000
158	17	158	15	0.0000	0.0000	0.0000	180.0000	0.0000	0.0000
158	17	158	17	0.0000	0.0000	0.0000	180.0000	0.0000	0.0000

Table S8: Partial Charge

Atom id	Partial Charge (e)
10	-0.5

45	1
107	-0.5

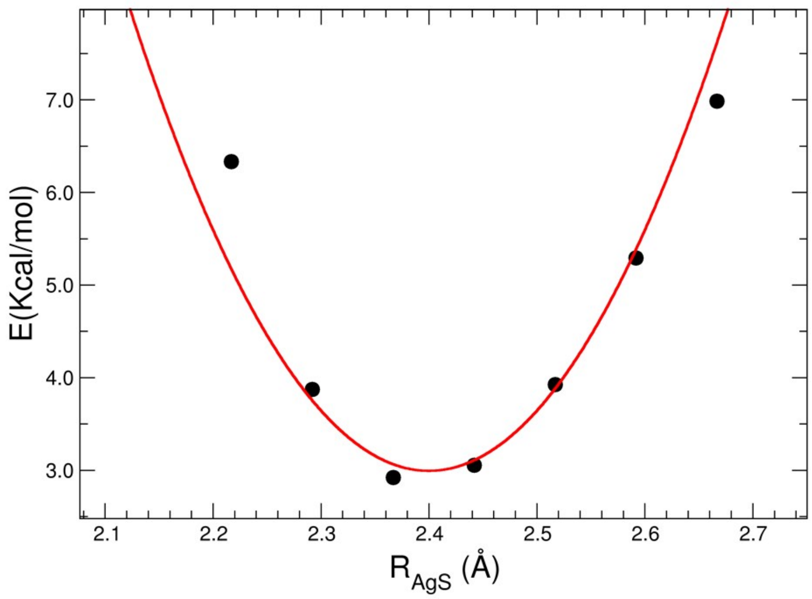

Figure S5: DFT potential energy scan for the L1 ligand along the Ag-S coordinate.

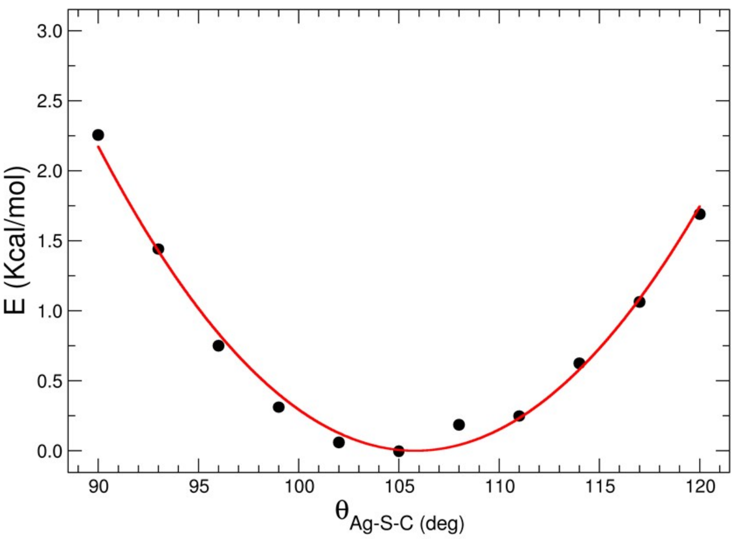

Figure S6: DFT potential energy scan for the L1 ligand along the Ag-S-C angle.

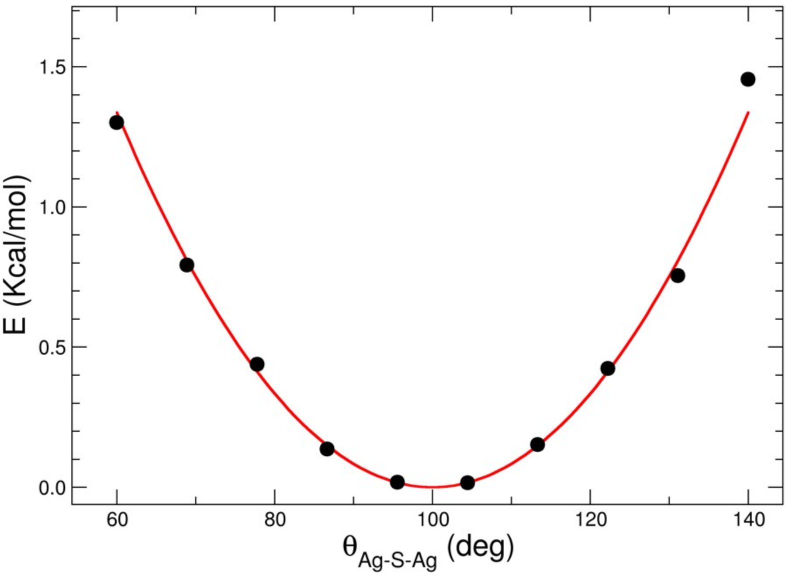

Figure S7: DFT potential energy scan for the L1 ligand along the Ag-S-Ag angle.

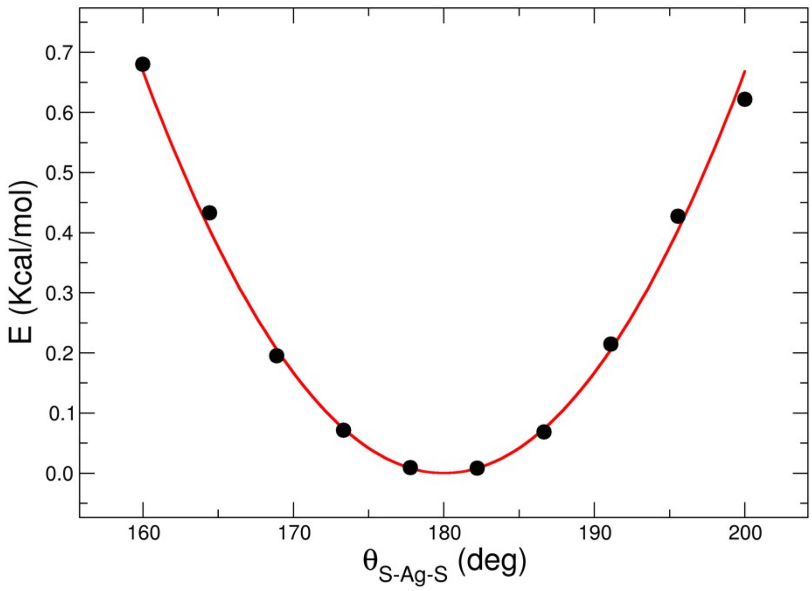

Figure S8: DFT potential energy scan for the L1 ligand along the S-Ag-S angle.

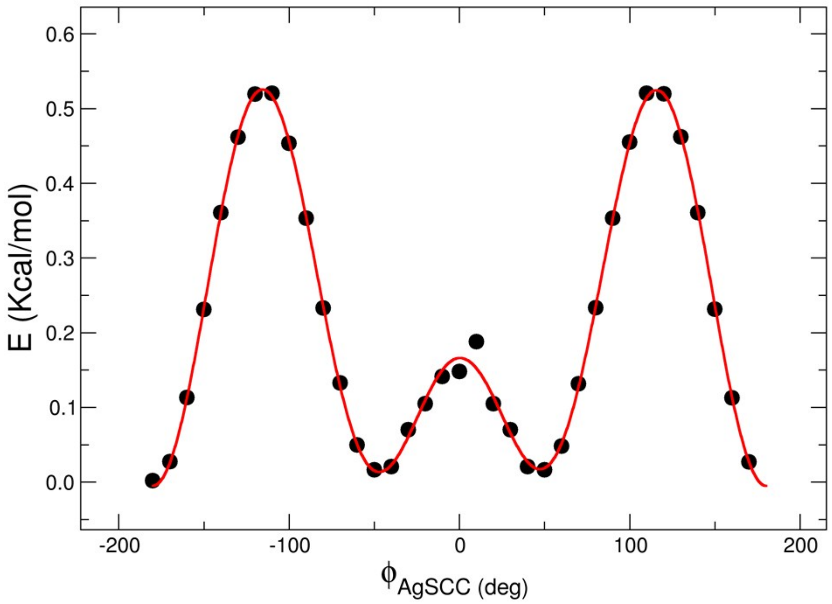

Figure S9: DFT potential energy scan for the L1 ligand along the Ag-S-C-C dihedral angle.
